# Smart Wireless Near‐Infrared Light Emitting Contact Lens for the Treatment of Diabetic Retinopathy

**DOI:** 10.1002/advs.202103254

**Published:** 2022-01-29

**Authors:** Geon‐Hui Lee, Cheonhoo Jeon, Jee Won Mok, Sangbaie Shin, Su‐Kyoung Kim, Hye Hyeon Han, Seong‐Jong Kim, Sang Hoon Hong, Hwanhee Kim, Choun‐Ki Joo, Jae‐Yoon Sim, Sei Kwang Hahn

**Affiliations:** ^1^ Department of Materials Science and Engineering Pohang University of Science and Technology (POSTECH) 77 Cheongam‐ro, Nam‐gu Pohang Gyeongbuk 37673 South Korea; ^2^ Department of Electrical Engineering Pohang University of Science and Technology (POSTECH) 77 Cheongam‐ro, Nam‐gu Pohang Gyeongbuk 37673 South Korea; ^3^ Department of Ophthalmology and Visual Science Seoul St. Mary's Hospital College of Medicine The Catholic University of Korea 505, Banpo‐dong Seocho‐gu Seoul 06591 South Korea; ^4^ PHI BIOMED Co. 168, Yeoksam‐ro Gangnam‐gu Seoul 06248 South Korea

**Keywords:** contact lens, diabetic retinopathy, light emitting diodes, prevention

## Abstract

Diabetic retinopathy is currently treated by highly invasive repeated therapeutic injections and surgical interventions without complete vision recovery. Here, a noninvasive smart wireless far red/near‐infrared (NIR) light emitting contact lens developed successfully for the repeated treatment of diabetic retinopathy with significantly improved compliance. A far red/NIR light emitting diode (LED) is connected with an application‐specific integrated circuit chip, wireless power, and communication systems on a PET film, which is embedded in a silicone elastomer contact lens by thermal crosslinking. After in vitro characterization, it is confirmed that the retinal vascular hyper‐permeability induced by diabetic retinopathy in rabbits is reduced to a statistically significant level by simply repeated wearing of smart far red/NIR LED contact lens for 8 weeks with 120 µW light irradiation for 15 min thrice a week. Histological analysis exhibits the safety and feasibility of LED contact lenses for treating diabetic retinopathy. This platform technology for smart LED contact lens would be harnessed for various biomedical photonic applications.

## Graphical abstract

A noninvasive smart wireless far red/near‐infrared (NIR) light emitting contact lens is developed for the repeated treatment of diabetic retinopathy. The diabetic retinopathy in rabbits is reduced to a statistically significant level by simple repeated wearing of smart far red/NIR light emitting diode contact lens for 8 weeks with 120 µW light irradiation for 15 min thrice a week.

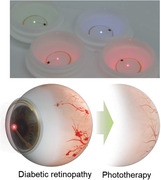

## 1 Introduction

Diabetes mellitus causes various complications including microvascular diseases of retinopathy, nephropathy, and neuropathy, macrovascular diseases of myocardial infarction, transient ischemic attacks and strokes, peripheral arterial disease, and immune dysfunction on cellular immunity.^[^ ^1^, ^2^ ^]^ Among them, diabetic retinopathy is the most common cause of adult blindness with initial retinal capillary microaneurysms. Currently, diabetic retinopathy is treated by laser irradiation,^[^ ^3^ ^]^ eye injection,^[^ ^4^ ^]^ and eye surgery.^[^ ^5^ ^]^ Laser treatment suppresses the progression of diabetic retinopathy by coagulating new blood vessels in the retina. In the case of eye injection, antiangiogenic drugs to inhibit the expression of new blood vessels in the retina, such as ranibizumab (Lucentis) and aflibercept (Eylea), are injected into the eye, preventing the progression of diabetic retinopathy. Eye surgery is generally performed to remove scars on the retina or blood vessels that cannot be removed by laser. These treatments are highly invasive and cause serious patient incompliance.

Recently, photobiomodulation has been extensively investigated for various light‐based therapy with therapeutic mechanism studies.^[^ ^6^, ^7^, ^8^, ^9^, ^10^ ^]^ Currently, light emitting diode (LED) masks and helmets are commercially available with food and drug administration (FDA) approvals. It is known to stimulate the cytochrome C oxidase in the mitochondria for the production of reactive oxygen species, adenosine triphosphate, and nitric oxide (NO), promoting skin rejuvenation^[^ ^11^, ^12^ ^]^ and hair growth.^[^ ^13^ ^]^ In addition, photobiomodulation has been applied to ocular disease treatment.^[^ ^14^, ^15^, ^16^, ^17^, ^18^ ^]^ The irradiation of light with a specific wavelength has shown a remarkable therapeutic effect on methanol toxicity,^[^ ^16^ ^]^ phototoxicity,^[^ ^17^ ^]^ and diabetic retinopathy.^[^ ^18^ ^]^ Unlike laser treatment, photobiomodulation has the advantage to be used in daily life by irradiating light with weak intensity to the eye.

Here, we develop a smart wireless LED contact lens for the prevention and intervention of diabetic retinopathy with a remotely controllable LED light dose. Recently, smart contact lenses are in the spotlight as a platform suitable for various healthcare applications^[^ ^19^, ^20^, ^21^, ^22^, ^23^, ^24^, ^25^ ^]^ including diabetic diagnosis,^[^ ^22^, ^23^ ^]^ intraocular pressure monitoring,^[^ ^24^ ^]^ and drug delivery.^[^ ^25^ ^]^ Our LED contact lens consists of four main parts: a far red/near‐infrared (NIR) LED (630–1000 nm), a resonant inductive wireless energy transfer system, an application‐specific integrated circuit (ASIC) chip with a power management unit, and a remote radio frequency (RF) communication system. The ocular far red/NIR LED treatment can partly replace invasive surgical treatments using specific wavelengths that are effective in preventing diabetic retinopathy. After in vitro characterization, we demonstrate the photobiomodulation effect of smart LED contact lens on diabetes‐induced retinal damage in rabbits with the analysis of glial fibrillary acidic protein (GFAP), vimentin, intercellular adhesion molecule‐1 (ICAM‐1), vascular endothelial growth factor (VEGF), complement 3 (C3), and cyclooxygenase‐2 (COX2) in the retina. Histological analysis and electroretinogram (ERG) are also performed to confirm the photobiomodulation effect.^[^ ^26^, ^27^, ^28^ ^]^

## 2 Results

### 2.1 Fabrication and Characterization of Smart Far Red/NIR LED Contact Lens

**Figure 1:**
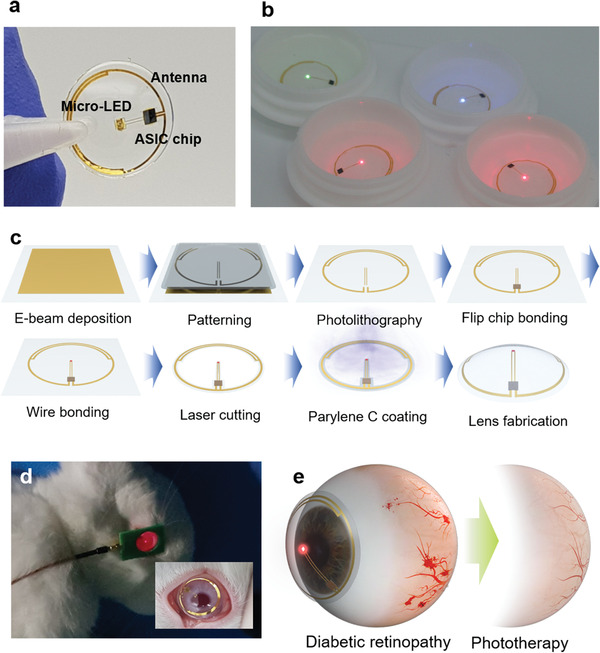
Schematic illustration for the preparation and phototherapy of smart LED contact lens. a) Photoimage of smart LED contact lens. b) Photoimage of wirelessly red green blue (RGB) color lighting LED contact lenses. c) Schematic illustration for the fabrication process of smart LED contact lens: E‐beam deposition of Cr/Au for thin film coating on the cleaned PET substrate, patterning by photolithography, ASIC chip bonding with a flip‐chip bonder, LED bonding with a wire bonder, laser cutting of the unnecessary part of PET substrate, Parylene C coating for passivation, and lens fabrication by curing in the silicone elastomer solution. d) Photoimage of the far red/NIR light irradiation to a rabbit eye to treat diabetic retinopathy. e) Schematic illustration of the diabetic retinopathy treatment using a smart LED contact lens.

As schematically shown in **Figure** **1**a, smart far red/NIR LED contact lens is fabricated by the chemical crosslinking of silicone elastomer solution to encapsulate a far red/NIR LED, an ASIC chip, and an antenna on a poly(ethylene terephthalate) (PET) film with the passivation of Parylene C. The transmitter coil, which is connected to a customized power amplifier, wirelessly transfers electrical power to the far red/NIR LED contact lens for light emission (Figure 1b). The colors of light can be varied by simply changing the type of LED in the contact lenses. After depositing Au on PET, the antenna and electrodes are patterned by the photolithography process with integrating the ASIC chip and LED to the electrode by flip‐chip bonding and wire bonding (Figure 1c). After removing the unnecessary part of PET film via laser cutting, whole devices are passivated by the deposition of Parylene C. Finally, smart LED contact lenses are fabricated to encapsulate the device using a silicone elastomer solution in a contact lens mold. The prepared smart LED contact lens is operated wirelessly on rabbit eyes to treat diabetic retinopathy (Figure 1d,e).

For the application of hydrophobic silicone elastomer as a contact lens material, we carried out the surface modification to reduce the contact angle with biocompatible and hygroscopic hyaluronic acid (HA) (Figure S1a, Supporting Information). Fourier transform ‐ infrared spectroscopy (FT‐IR) analysis confirmed the silicone elastomer surface modification with HA‐NH_2_ (Figure S1b, Supporting Information). Most of the contact lenses have a contact angle around 30°–40° for commercial applications. First, the surface of silicone elastomer contact lens was treated by O_2_ plasma to introduce functional groups. Then, (3‐aminopropyl)triethoxysilane (APTES) was reacted with the functional groups to introduce amine groups on the surface, which was reacted with glutaraldehyde to introduce aldehyde groups on the contact lens surface. After that, the contact lens was reacted with HA‐NH_2_ for the surface modification of silicone elastomer with HA (Figure S1a, Supporting Information). The contact angle of contact lens surface‐treated with HA‐NH_2_ increased from about 36° to 60° with increasing time for up to 60 d, which was significantly lower than 116° of the contact lens without surface treatment (Figure S1c, Supporting Information). In the case of surface‐treated contact lenses, the oxygen permeability was 101.91 Dk, which was lower than that of the control contact lenses (146.24 Dk), but higher than that of commercially available contact lenses (Figure S1d, Supporting Information). From the results, the surface modified silicone elastomer contact lens might be used for further applications.^[^ ^29^, ^30^ ^]^ The visible light transmittance of the surface‐treated contact lens was more than 90%, which was adequate for practical applications (Figure S1e, Supporting Information).

### 2.2 Controller ASIC Chip for Wireless Powering and μLED Lighting

The designed wireless power transfer system leverages a resonant inductive coupling. The antenna at the external reader acts as the first coil. It wirelessly transmits energy to the secondary coil which is a loop antenna fabricated on the contact lens. The resonance frequency is determined by the equivalent inductance, *L*, of the secondary coil and the total capacitance connected in parallel to the *L*. The capacitance consists of input capacitance of ASIC chip and parasitic gold stud bumps for flip‐chip bonding. The load impedance observed from the first coil becomes the highest at the resonance frequency, resulting in the maximum efficiency in the energy transfer.

The ASIC chip rectifies the incoming RF energy and generates an internal supply voltage, *V*_DD_ (Figure S2a, Supporting Information). A reference voltage (*V*_REF_) generator, whose characteristics are robust to supply variations, provides a bias voltage to flow a constant current through an on‐chip resistor. The constant current becomes a reference current to be copied to the μLED driver. This reference current settles after a startup circuit turns on. The startup is triggered when the incoming RF energy increases above a given threshold. A voltage limiter is also added to prevent the circuit from possible breakdown due to excessively charged *V*_DD_ by RF overdriving. The power dissipated by the ASIC chip is 19.6 µW at *V*_DD_ of 3.3 V. The ASIC chip, fabricated by 180 nm complementary metal oxide semiconductor (CMOS) process, occupies an area of 1.0 mm × 1.0 mm. The thickness of the chip is made to be 150 µm by backside grinding. A photoimage is shown in Figure S2b in the Supporting Information.

To evaluate the power conversion efficiency (PCE) of the ASIC chip, the delivered power is also measured to *V*_DD_ at a distance of 1 cm, while the network analyzer drives the first coil with 50 Ω matched. This measurement corresponds to the combined result of power transfer efficiency (PTE) and PCE (Figure S2c, Supporting Information). While our design target for the resonant frequency was 433 MHz, the highest efficiency was observed at 470 MHz due to the shift in the fabricated resonant frequency in this work. However, within the distance of 1 cm, the delivered power at 433 MHz was still sufficient enough to perform the required operation for smart contact lenses. The PTE between the two coils was shown at 433 MHz with increasing distance in Figure S2d in the Supporting Information. Since the power transfer system was set to generate 433 MHz, the overall wireless power could be further improved by adding more capacitance in the ASIC chip to downshift the resonant frequency to 433 MHz.

The custom‐made external RF power transmission board was designed for smart LED contact lenses for in vivo phototherapy and implemented on a flexible printed circuit board. This external board consisted of a commercial RF power transmitter (MAX41460), a field‐programmable gate array (FPGA) processor to RF control, and an external loop antenna with a matching network (Figure S3, Supporting Information). A Bluetooth module was implemented for the control with a smartphone device. The RF power and transmission frequency could be controlled in 1 MHz increments in the FPGA processor in detail and operated with a laptop or a smartphone.

### 2.3 Optical Properties of Smart LED Contact Lens

**Figure 2:**
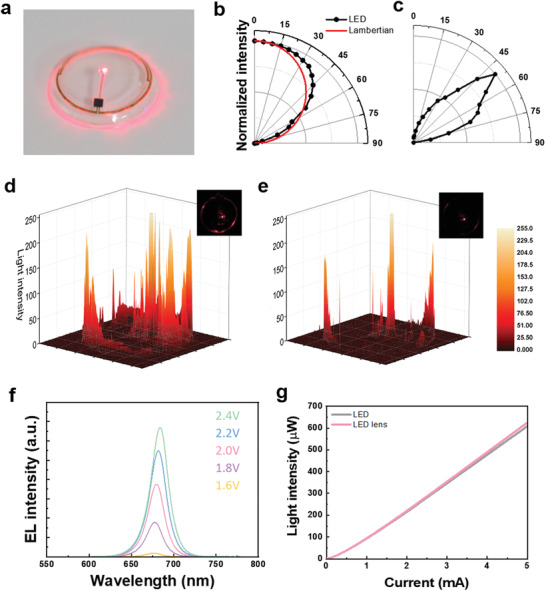
Optical characteristics of smart LED contact lens. a) Photoimage of smart LED contact lens. Angle dependent light intensity of smart LED contact lens: b) central part of smart LED contact lens and c) edge part of smart LED contact lens. Light intensity analysis by using image J and photoimages of smart LED contact lenses d) in the air and e) in PBS. f) electroluminescent (EL) intensity of smart contact lens with increasing wavelength and voltage. g) The light intensity of LED and smart LED contact lens with increasing current (*n* = 9).

As reported in refs. [14, 15, 18], we used the wavelength of ≈670 nm to treat diabetic retinopathy for the far red/NIR micro‐LED in our smart contact lenses. The size of LED was around 325 µm^2^ and the thickness was around 150 µm. The LED was connected with the ASIC chip for the power supply via the antenna (**Figure** **2**a). The LED was placed at the center of the contact lens for light delivery to the retina. The transmittance of silicone elastomer, the material of the contact lens, was about 90% in the wavelength of 300–800 nm (Figure S1e, Supporting Information). At the center of the contact lens, light was spread like Lambertian distribution (Figure 2b), but the edge part of the contact lens showed the highest intensity at the angle of 50° (Figure 2c). The intensity of the LED light was 40 µW at 880 µA, 80 µW at 1440 µA, 160 µW at 2640 µA, and 320 µW at 4880 µA. While the light from the LED was lost to the edge of the contact lens by total reflection in the air, most of the light was transmitted through the center in the aqueous environment of phosphate buffered saline (PBS) (Figure 2d,e and Figure S4, Supporting Information). From the results, all of the light emitted from the LED contact lens might be transferred to the retina. The peak wavelength of the LED electroluminescent was 675 nm at 1.8 V (Figure 2f) with LED intensity of 116 µW at 1.2 mA (Figure 2g). The current density and luminesce of μLED was 6651 mA cm^−2^ and 2041 cd m^−2^, respectively (Figure S5ab, Supporting Information).

### 2.4 In Vivo Safety of LED Contact Lens

**Figure 3:**
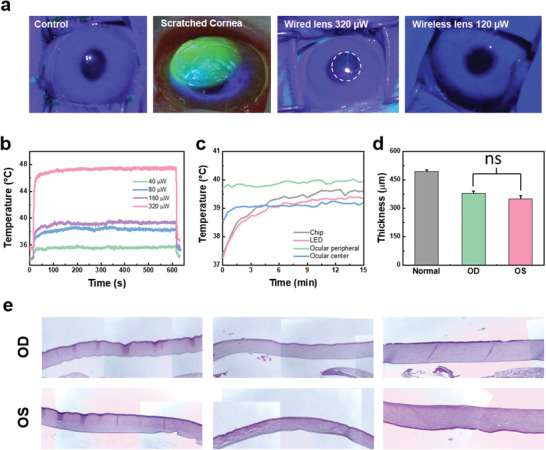
In vivo safety assessment of smart LED contact lens. a) Corneal thermal damage analysis by fluorescein staining for the normal control cornea, scratched cornea, 320 µW light treated cornea using a wired smart LED contact lens, and 120 µW treated cornea using a wireless smart LED contract lens. b) The temperature change of the LED with increasing light intensity and irradiation time. c) The temperature of LED, chip, and ocular peripheral and center with increasing irradiation time of smart wireless 120 µW LED contract lens (*n* = 42). d) Corneal thickness of diabetic rabbits: OD with LED treatment and OS without LED treatment (*n* = 3, *P* > 0.05, not significant). e) optical microscopic (OM) images of stained diabetic corneas.

The safety of smart far red/NIR LED contact lens was evaluated in the eyes of New Zealand white rabbits for 8 weeks. The safety evaluation was focused on the corneal damage by the heat generated during the operation of smart LED contact lens. The corneal damage was assessed by corneal staining with fluorescein. When the cornea is damaged, it shows green fluoresce. The rabbit's cornea that received 40, 80, and 160 µW of light was intact, but the rabbit's cornea that received 320 µW of light showed slight green fluorescence due to the thermal damage (**Figure** **3**a and Figure S6, Supporting Information). When 40 µW of light was applied to the cornea using an LED contact lens, the LED temperature rose to 35.6 °C, 80 µW of light to 38.6 °C, and 160 µW of light to 39.5 °C. When the light intensity increased to 320 µW, the LED temperature was 47.4 °C (Figure 3b).

Based on these results, wireless LED contact lenses were assessed for 8 weeks with irradiation of 120 µW of light for 15 min. The temperature of eyeballs wearing wireless contact lenses increased over time, but did not rise to the point of damaging the eyeball. The temperature of the center and the peripheral side of rabbit's eyeballs were in the range of 38–40 °C, and the temperature increased by the chip and the LED was about 39 °C (Figure 3c). Since the wireless LED contact lens used a low amount of light, there was no observed corneal damage in the safety evaluation with fluorescein. From the results, we could confirm the safety of smart LED contact lens without significant heat generation by LEDs and chips.

In addition, we checked the safety of smart LED contact lenses by measuring the corneal thickness. The cornea is known to become thick in the presence of inflammatory reaction by the corneal damage. In the rabbit eye safety experiment using wired LED contact lenses, corneal thickness in rabbits did not change (Figure S7, Supporting Information). As shown in Figure 3d, oculus dextrus (OD) was an ocular treated with light using an LED contact lens, and oculus sinister (OS) was an ocular without treatment as a control. Since there was no difference in the corneal thickness between OD and OS, we could confirm the safety of LED and chip in the contact lens without significant corneal inflammatory reaction (Figure 3e). In addition, since there was no difference in the tear volume without and with lens‐wearing, the prolonged contact lens wearing appeared not to cause dry eye syndrome (Figure S8, Supporting Information).

### 2.5 In Vivo Therapeutic Application of Smart Contact Lens

Before in vivo experiments, the effect of far red/NIR light irradiation was assessed on the viability of adult retinal pigment epithelial cells (ARPE‐19) in a hyperglycemic environment (Figure S9, Supporting Information). Remarkably, the cell viability increased with increasing voltage, showing a positive effect on the cell growth. To find the optimal phototherapy condition for diabetic retinopathy, we first performed in vivo wired LED contact lens experiments in six groups of diabetic rabbits for 8 weeks: a positive control, a negative control, 40, 80, 160, and 320 µW light‐treated groups. Each retina was analyzed by Western blot to assess the effect of light intensity on preventing diabetic retinopathy (Figure S10, Supporting Information). The therapeutic effect was determined by measuring six different biomarkers of GFAP, vimentin, ICAM‐1, VEGF, C3, and COX2. GFAP and vimentin, related to diabetic retinopathy and stress, were the lowest for the case of 160 µW treatment at the value of 2.607 and 0.909. C3 is related to inflammation and tissue damage, which was also the lowest value (1.963) for the case of 160 µW treatment. The main symptom of diabetic retinopathy is the leakage of blood from the loosened blood vessels and related with COX2, ICAM‐1, and VEGF. Accordingly, the inhibition of angiogenesis is vital for diabetic retinopathy. After 160 µW treatment for 8 weeks, angiogenic factors were dramatically decreased from 2.602, 2.618, and 1.810 to 1.108, 1.818, and 1.415, respectively. All these results confirmed that the biomarkers of diabetic retinopathy were reduced by phototherapy with smart far red/NIR LED contact lenses. In addition, immunohistochemical analysis showed the best efficacy when treated with 160 µW of light for 8 weeks (Figures S11 and S12, Supporting Information). The optimal therapeutic effect of the far red/NIR LED contact lens treatment was obtained with a light intensity of 160 µW for 600 s (96 mJ). Based on these results and the safety assessment, we decided to perform the wireless LED contact lens treatment with a light intensity of 120 µW for 900 s (108 mJ). Although the rabbits were anesthetized, their pupils were moving during the experiment and the wireless energy transmission was reduced by 5–10% compared to the highest efficiency. Accordingly, the wireless phototherapy would be better to be performed under the condition of 120 µW for 900 s (108 mJ), which was ≈10% more than 160 µW for 600 s (96 mJ).

**Figure 4:**
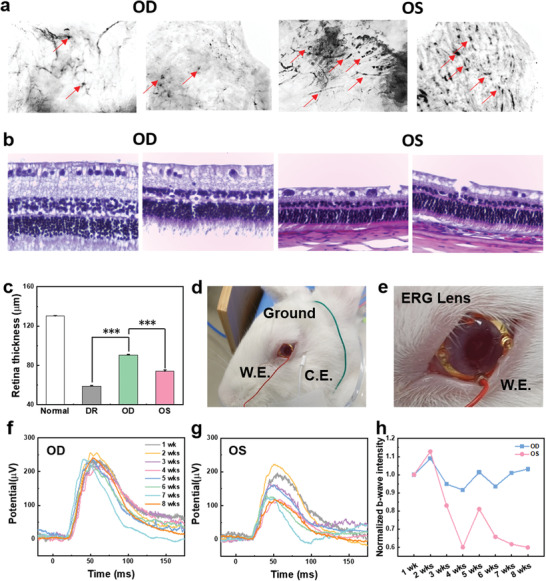
In vivo phototherapeutic effect of smart LED contact lens. a) ADPase stained diabetic retina images and b) histological images for the retina thickness before and after phototherapy: OD with LED treatment and OS without LED treatment. c) Retina thickness for the normal, diabetic retinopathy (DR), OD, and OS (*n* = 3, ***P < 0.0005). The photoimages of d) the rabbit and e) the magnified eye for ERG analysis. The ERG signals for 8 weeks f) with and g) without far red/NIR LED contact lens treatment. h) The relative b‐wave intensity of OD and OS for 8 weeks.

**Figure** **4**a shows the stained image of retinal blood vessels with adenosine diphosphatase (ADPase), exhibiting the spots of neovascularization or hemorrhage. There were fewer spots in OD with phototherapy than in OS without phototherapy (Figure S13, Supporting Information). In the case of diabetic retinopathy, rapid neovascularization in the retina increases blood permeability in blood vessels, resulting in bleeding in the retina. The photobiomodulation therapy with smart LED contact lenses appeared to significantly reduce the neovascularization and hemorrhage in the retina. In addition, when diabetic retinopathy occurs, the optic nerve of the retina is damaged with the decreased thickness of the retina. As shown in Figure 4b,c, the retinal thickness of OD with phototherapy was 90.63 µm, whereas that of the OS without phototherapy was only 74.11 µm (Figure S14, Supporting Information). These results confirmed the therapeutic effect of smart LED contact lenses on the retinal damage. The ERG signal was measured once a week for 8 weeks to analyze the intensity of b‐waves related to diabetic retinopathy (Figure 4d,e). The b‐wave intensity did not change in the treated eye of OD for 8 weeks (Figure 4f), whereas the b‐wave intensity was decreased in the untreated eye of OS (Figure 4g). Figure 4h compares the normalized intensity of b‐wave signals over time between treated OD and untreated OS. From all these results, we could confirm the meaningful preventive effect of far red/NIR light therapy using smart LED contact lenses on diabetic retinopathy.

Figure S15 in the Supporting Information shows the inflammation in OD and OS by the dot blotting analysis of vitreous for the expression levels of VEGF, ICAM‐1, vascular cell adhesion molecule 1 (VCAM‐1), interleukin‐6 (IL‐6) and interleukin‐8 (IL‐8). The vascular‐related factors of VEGF, VCAM‐1, and ICAM‐1 were reduced by the treatment with wireless LED contact lens. In addition, IL‐6 and IL‐8, which are related to inflammation, also showed the trend of decrease when treated with a far red/NIR LED contact lens. Although the dot blot analysis is generally used for semiquantitative analysis, we could confirm the therapeutic trend of wireless LED contact lens.

**Figure 5:**
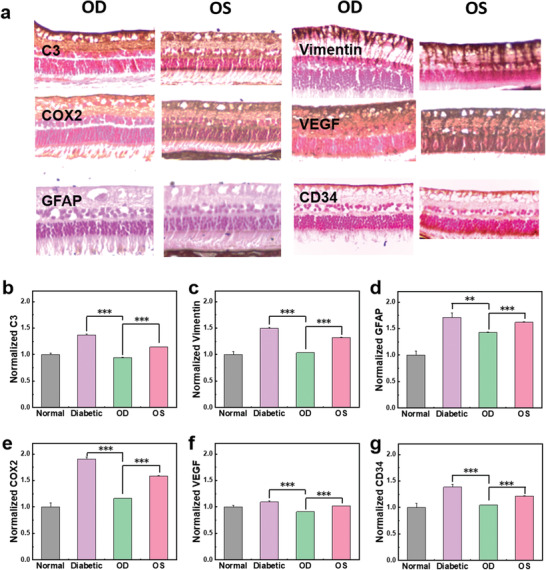
Immunohistochemical analyses for the phototherapy using a smart LED contact lens. a) Immunohistochemical images of retinas for C3 related to inflammation and tissue damage, vimentin and GFAP related to diabetic retinopathy and stress, COX2, ICAM1, and VEGF related to angiogenesis: OD with wireless LED treatment and OS without LED treatment. The normalized quantification of the immunohistochemical images by image J analysis: b) C3, c) vimentin, d) GFAP, e) COX2, f) VEGF, and g) CD34 expression on the retina (*n* = 3, **P < 0.005, ***P < 0.0005).

Furthermore, there was significant difference between the immunohistochemically stained images of retinas in diabetic rabbits after treatment with (OD) and without (OS) wireless LED contact lenses (**Figure** **5** and Figures S16–S18, Supporting Information). The photobiomodulation therapy in OD reduced the expression of C3, vimentin, cluster of differentiation (CD) 34, COX2, VEGF, and GFAP significantly in comparison with OS. C3, associated with inflammation and tissue damage, was remarkably reduced by photobiomodulation. In addition, vimentin and GFAP, related to diabetic retinopathy and retina stress, were significantly decreased in the treated group with the far red/NIR LED contact lens compared to the untreated group. COX2, VEGF, and CD34, factors related to the expression of angiogenesis, were also notably reduced in the treated group. The protein expression in the retina revealed the effect of smart LED contact lens on preventing diabetic retinopathy.

## 3 Discussion

Smart contact lenses have attracted great attention for healthcare applications especially for diagnostic applications including continuous glucose monitoring for diabetes and intraocular pressure monitoring for glaucoma. In contrast, there are few reports on the therapeutic applications of smart contact lenses. Recently, we successfully developed a wireless smart contact lens for diagnostic and drug delivery applications for the treatment of diabetic retinopathy.^[^ ^22^ ^]^ The loaded drugs in drug reservoirs with gold (Au) membrane sealing could be released by applying electrical current for the dissolution of Au to AuCl_4_^−^. However, the amount of drugs loaded in the smart contact lens was limited, making it difficult for long‐term repeated therapy. In this work, we developed smart far red/NIR LED contact lenses which could be used for the prevention and intervention of diabetic retinopathy for a long‐term period. The therapeutic effect of smart far red/NIR LED contact lens was successfully demonstrated from the reduced retinal vascular hyper‐permeability in rabbits with diabetic retinopathy. Histological analysis and ERG signal analysis also confirmed the safety and feasibility of LED contact lenses for treating diabetic retinopathy. In comparison with conventional repeated drug injections and surgical interventions, the phototherapy using a smart LED contact lens is noninvasive and can be used repeatedly in any place for a long‐term period. In addition, it is possible to control the quantitative amount of light according to the disease status of diabetic patients. Table S1 in the Supporting Information describes the purpose, pros and cons of our smart LED contact lens in comparison with the conventional surgical intervention and laser treatment.

Although the mechanism for the prevention of diabetic retinopathy by photobiomodulation is not clear yet, there are several proposed hypotheses. In diabetic retinopathy, the capillaries of the retina are damaged by high blood sugar with constricted capillaries. The ischemic tissue, which has become difficult to receive oxygen due to the damaged capillaries, generates angiogenesis factors and new blood vessels are abnormally formed even to the retina. Because the abnormally generated blood vessels do not form a blood‐retina barrier, blood and fat leak into the retina, leading to the damage in eyesight.^[^ ^31^ ^]^ However, far red/NIR light with a wavelength of 670 nm plays an important role in dilating blood vessels. NO is generated by endothelium cells rather than endothelial nitric oxide synthase (eNOS) upon far red/NIR light irradiation. The NO generated at this time is a small amount, but sufficient enough to dilate blood vessels. For this reason, the occurrence of new blood vessels is likely to be prevented in the retina treated with far red/NIR light. In other words, the ischemic tissue may not occur due to the dilation of the capillaries of the retina.^[^ ^32^ ^]^ Although the intervention mechanism of diabetic retinopathy by using far red/NIR light requires further research, this study is of great significance as it demonstrates that smart far red/NIR contact lenses can prevent diabetic retinopathy.

Concerning the safety issue of smart LED contact lens, the possible ocular damage should be carefully investigated by the generated heat from the wireless power transfer system and LED. In this context, we analyzed the heat generated by operating smart LED contact lens using a thermal infrared camera. Although the temperature increase on the rabbit eyes was notable for the case of 320 µW light intensity from the wired smart LED contact lens, the corneal damage by heat was not significant with the temperature increase to 39.5 °C for the case of 160 µW light intensity (Figure 3b). Accordingly, we used wireless smart LED contact lenses with irradiation of 120 µW light for 15 min for 8 weeks. Optical and histological analyses of corneas in the eyes of New Zealand white rabbits also confirmed the safety of smart LED contact lens.

In summary, a smart far red/NIR LED contact lens has been successfully developed with a micro‐LED controlled by wireless power and remote communication systems for on‐demand phototherapy on diabetic retinopathy. While the smart LED contact lens showed the total reflection of light in the air, most of the light was delivered from the contact lens in the aqueous environment. The safety of the smart far red/NIR LED contact lens was confirmed by measuring the heat generated during the operation of LED and the corneal thickness change in the eyes of New Zealand white rabbits for 8 weeks. Furthermore, the therapeutic effect of smart LED contact lens was confirmed by analyzing six different biomarkers of GFAP, vimentin, ICAM‐1, VEGF, C3, and COX2, the retinal thickness and the ERG signals. Although the exact mechanism for the photobiomodulation effect on diabetic retinopathy was not clearly proved yet, we could observe a lot of evidence for the feasibility of our smart LED contact lens on diabetic retinopathy. This smart LED contact lens would be harnessed as a next‐generation wearable device to achieve the on‐demand medication for ubiquitous healthcare applications to various ocular and other diseases using different light sources.

## 4 Experimental Section

### Overall Fabrication of Integrated Smart Contact Lens

A power receiver coil and an IC chip were placed on the peripheral area of a contact lens to keep the pupil area open. The electrode of the wireless lighting device was fabricated by Au deposition via e‐beam evaporation at a thickness of 500 nm on an ultrathin PET film (Mitsubishi, 25 µm) and patterned by using the photolithography to make a pad and coils for the connection to the LED electrodes and the ASIC chip. The ASIC chip was implemented through the standard 0.18 µm CMOS process and diced into the dimension of 1.0 mm × 1.0 mm × 0.15 mm by chemical polishing and mechanical sawing. The ASIC chip was integrated into wireless lighting devices by using a flip‐chip bonder and LED (Epigap, EOLC‐670‐15) was connected by using a wire bonder. For the insulation and waterproof, all devices on the PET substrate were coated with Parylene C. The wireless lighting device was embedded in the soft contact lens using a silicone elastomer (Nusil, MED‐3015) contact lens solution and a mold. MED‐3015 originally has about 90–95% transparency in the visible region. After curing the contact lens, the smart far red/NIR LED contact lens was treated with O_2_ plasma for surface modification.

### Surface Treatment of LED Contact Lens

After fabricating the silicone elastomer contact lens, the surface was treated with O_2_ plasma at 100 W and 100 sccm for 5 min. After that, the contact lens was put into a solution of 10 wt% APTES at 56 °C for 2 h. Then, the contact lens was rinsed with deionized (DI) water more than 3 times and dried with N_2_ gas. Next, the contact lens was put into 2.5 wt% of glutaraldehyde at room temperature for 1 h and rinsed with DI water. Glutaraldehyde treated contact lens was put into 10 mg mL^−1^ of HA‐NH_2_ (MW = 10 kDa) solution for 6 h and rinsed with DI water.

### Characterization of LED Contact Lens

The brightness and spectrum of an LED contact lens were analyzed with a spectroradiometer (CS 2000, Konica Minolta sensing). The intensity of the light was measured with a photodiode power sensor (S120VC, Thorlabs) and the angle dependence intensity was measured with a spectroradiometer (CS 2000, Konica Minolta sensing). The LED thermal emission was measured with a thermal imaging camera (A325SC, FLIR) and analyzed by using FLIR tools.

### Power Transmission Efficiency Measurement

The wireless power transmission system was prepared with a transmitter coil, a receiver coil on a contact lens, a function generator (AFG 3101, Tektronix), a commercial power amplifier module (MAX 7060) and an ASIC chip. The power amplifier module transferred energy to the receiver coil via the transmitter coil. To evaluate the performance of the wireless power transfer between two coils, the ratio between the received and the transmitted powers was measured at the first and the second coils, respectively. The ratio was defined to be the PTE using a network analyzer (N5230A, Agilent).

### In Vitro Photobiomodulation

ARPE‐19 cells were incubated in a dark incubator with Dulbecco's modified eagle medium (DMEM): F‐12 medium containing 5 × 10^−3^ m glucose, 10% fetal bovine serum, and 1% antibiotic‐antimyotic (AA). For in vitro photobiomodulation, ARPE‐19 cells were seeded in the black 96 multiwell plates and incubated in a dark incubator with 5 or 30 × 10^−3^ m glucose in the cell culture media (DMEM: F‐12). The photobiomodulation was performed for 30 × 10^−3^ m glucose of ARPE‐19 cells with an LED array. The plates were irradiated with an LED array of 1.45, 1.5, 1.6, and 1.7 V for 6 min. The other plate was kept away from light to compare the photobiomodulation effect. The treatments were performed twice a day for 3 d.

### Preparation of Diabetic Retinopathy Model Rabbits

For in vivo treatment of diabetic retinopathy, alloxan‐induced diabetic model rabbits were prepared by a single injection of 150 mg kg^−1^ alloxan to New Zealand white rabbits (2.8–3.8 kg) via the ear vein after fasting for 24 h. After that, the rabbits with a plasma glucose concentration higher than 140 mg dL^−1^ were considered to be diabetic.

### In Vivo Photobiomodulation Using Wired LED Contact Lens

New Zealand white rabbits were treated by Alloxan injection to induce diabetes. After one week, in vivo photobiomodulation treatment was performed with wired LED contact lenses using two control groups (normal and diabetic rabbits without treatment) and four experimental groups (wired LED contact lenses with an intensity of 40, 80, 160, and 320 µW) for 8 weeks. The photobiomodulation treatment was performed 10 min a day and 3 d a week in a dark room. The thermal damage of the cornea was analyzed by fluorescein staining and the therapeutic effect was assessed by the Western blotting (AE‐6530 mPAGE, ATTO). The Western blotting was conducted with the protein concentration of 2 mg mL^−1^ at 100 V for more than 2 h.

### In Vivo Photobiomodulation Using Smart Wireless LED Contact Lens

New Zealand white rabbits were treated by Alloxan injection to induce diabetes. After one week, in vivo photobiomodulation treatment was performed with smart wireless LED contact lenses using two control groups (normal and diabetic rabbits without treatment) and one experimental group (smart wireless LED contact lens with an intensity of 120 µW) for 8 weeks. The photobiomodulation treatment was performed 15 min a day and 3 d a week in a dark room. The thermal damage of the cornea was analyzed by fluorescein staining and the therapeutic effect was assessed by the dot blotting, ERG, and immunostaining.

### In Vivo Electroretinogram

New Zealand white rabbits were treated by Alloxan injection to induce diabetes. After one week, the ERG was performed once a week for 8 weeks. After fixing the rabbits anesthetized with Alfaxan and Rompun in an acrylic cage, the ground electrode was connected to the forehead and the counter electrode was connected between the eyes and ears. After wearing a contact lens with a working electrode, the ERG signal was measured with stimulating the optic nerve of the retina using light of 10 mcd.

### Schirmer Tear Test

Tear production was assessed by using the Schirmer Tear Test Standardized Sterile Strips (EagleVision, US). The Schirmer strip was applied on the temporal tarsal conjunctiva of the lower lid of the rabbit and maintained in place for 5 min. The volume of secreted tear was recorded by the advancement of blue dye on the marked standardized scale.

### Corneal Fluorescein Staining

The cornea was stained with fluorescein paper strips (Fluorescein Paper Strips – 0.4 m disodium fluorescein/DI water, Haag‐Strei Diagnositecs) soaked in a drop of 0.5% alkyne. After 5 s poststaining, it was washed with saline solution. The detailed examination of the corneal ulcer was performed by using a portable Slit‐Lamp Microscope (Keeler, UK). The ratio of staining was observed on the cornea with and without fluorescein staining.

### Histopathological and Immunohistochemical Analysis

Formalin‐fixed whole eyes were embedded in paraffin and 5 µm sections were prepared for the examination with hematoxylin and eosin (H&E) staining (Abcam, UK). Briefly, the immunohistochemical detection included antigen retrieval in 10 × 10^−3^ m citrate buffer in a microwave oven and blocking endogenous peroxidase with 1% hydrogen peroxide. Tissues were incubated at 4 °C overnight with the following primary antibodies; Complement 3 (sc‐28294, Santa Cruz), VEGF (sc‐7269, Santa Cruz), COX2(sc‐1745, Santa Cruz), ICAM‐1 (sc‐8439, Santa Cruz), GFAP (sc‐51908, Santa Cruz), vimentin (sc‐373717, Santa Cruz), and CD 34 (sc‐19621, Santa Cruz). Then, the Vectastain Elite avidin‐biotin complex (ABC) system (Vector Laboratories, Burlingame, CA) for horseradish peroxidase was used for immunohistochemistry. The tissues were lightly counterstained with nuclear fast red (Abcam, UK).

### ADPase Analysis for Neovascularization and Hemorrhage

Eyeballs were fixed with 4% paraformaldehyde for 24 h. After the cornea and lens were removed, the entire retina was dissected and radially cut into four quadrants. The retinas were washed in tris maleate buffer (pH 7.2, Sigma) on ice and put in a solution containing 5 mg adenosine diphosphate (ADP) (Sigma A2754), 21 mL of 0.1 m tris maleate buffer, 0.05 m sucrose (Sigma), 2.5 mL of 5% magnesium sulfate (Sigma) and 0.75 mL of 5% lead nitrate (Sigma). After incubation, the retina was developed with 0.01% ammonium sulfide (Sigma).

### Dot Blotting

The rabbits were enucleated and the eyes were stored at 20 °C. After thawing, the vitreous was centrifuged at 13 000 × *g* at 4 °C for 15 min to clear the samples from cellular debris. The concentrations of the vitreous were measured using the Bicinchoninic Acid Protein Assay Kit (Pierce, Thermo Scientific) according to the manufacturer's instruction. Three mg of total protein from the vitreous sample was spotted to poly(vinylidene fluoride) or poly(vinylidene difluoride) (PVDF) membrane and then the PVDF was dried and treated as described for Western blot analysis with the following primary antibodies: ICAM‐1 (sc‐8439, Santa Cruz), VEGF (sc‐7269, Santa Cruz), VCAM‐1 (sc‐8304, Santa Cruz), IL6 (ab‐6672, ABCAM) and IL8 (sc‐7922, Santa Cruz). The signals were visualized by using the enhanced chemiluminescence (ECL) Substrates for High‐Sensitivity Western Blot Detection (Bio‐Rad). The dot blots were analyzed and quantified by using Image J.

### Statistical Analysis

One‐tail statistical analysis was carried out using Student's *t*‐tests and extracted *P*‐values. Data are presented as means ± standard error of the mean (SEM). The difference of *P* < 0.05 was considered statistically significant (ns: *P* > 0.05, *P < 0.05, **P < 0.005, ***P < 0.0005). Statistical data analysis was performed by OriginPro (Origin 2019, OriginLab Inc.). Digital images of the light intensity, fluorescence signal, and the thickness of the ouclar tissue were quantified with ImageJ National Institutes of Health (NIH) for Figures 2d,e, 3e, 4b, and 5a.

### Study Approval

All in vivo animal experiments were conducted in accordance with the Association for Research in Vision and Ophthalmology (ARVO) Statement for the Use of Animals in Ophthalmic and Vision Research. The animal experiment was approved by the Institutional Animal Care and Use Committee (CRONEX‐ 202012002) at the CRONEX.

## Conflict of Interest

The authors declare no conflict of interest.

## Author Contributions

G.‐H.L. and C.J, contributed equally to this work. S.K.H. conceived and supervised the project, designed experiments, interpreted data, and wrote the paper. G.‐H.L. performed experiments, collected samples, analyzed and interpreted data, and wrote the paper. C.J., S.S., S.‐K.K., S.H.H., and J.‐Y.S. contributed to preparing and designing the LED contact lens. J.W.M., H.K., H.H.H., S.‐J.K., and C.‐K.J. contributed to designing and performing animal experiments. All authors contributed to the critical reading and revision of the paper.

## Code Availability

A full code availability statement is included in the paper. Custom algorithms are ancillary, but a full code is available in GitHub as a project name “LED_SCL_2021.” The authors used commercial software programs of Xilinx Integrated Synthesis Environment (ISE) Design Suite (ver.14.7) and Java (ver.1.8.0_131). The LSK_v1.v is a Verilog code used in FPGA board to read data from RF receiver. The guimake4.java is a java code to receive and plot data from the FPGA board on the computer.

## Supporting information

Supporting Information

## Data Availability

The data that support the findings of this study are available in the supplementary material of this article.
