# Supplementary material for: Smart Wireless Near‐Infrared Light Emitting Contact Lens for the Treatment of Diabetic Retinopathy
Source: Adv Sci (Weinh). 2022 Jan 29;9(9):2103254. doi: 10.1002/advs.202103254 (PMC8948592; doi:10.1002/advs.202103254)
Supplement: Supplementary file 1 — Supporting Information [file ADVS-9-2103254-s001.pdf]

ADVANCED SCIENCE
Open Access

Supporting Information

for *Adv. Sci.*, DOI 10.1002/advs.202103254

Smart Wireless Near-Infrared Light Emitting Contact Lens for the Treatment of Diabetic Retinopathy

*Geon-Hui Lee, Cheonhoo Jeon, Jee Won Mok, Sangbaie Shin, Su-Kyoung Kim, Hye Hyeon Han, Seong-Jong Kim, Sang Hoon Hong, Hwanhee Kim, Choun-Ki Joo, Jae-Yoon Sim and Sei Kwang Hahn**

ADVANCED SCIENCE
Open Access

Supporting Information

for *Adv. Sci.,* DOI: 10.1002/advs.202103254

Smart Wireless Near-Infrared Light Emitting Contact Lens for the Treatment of Diabetic Retinopathy

*Geon-Hui Lee, Cheonhoo Jeon, Jee Won Mok, Sangbaie Shin, Su-Kyoung Kim, Hye Hyeon Han, Seong-Jong Kim, Sang Hoon Hong, Hwanhee Kim, Choun-Ki Joo, Jae-Yoon Sim, and Sei Kwang Hahn**

Supporting Information

Smart Wireless Near-Infrared Light Emitting Contact Lens for the Treatment of Diabetic Retinopathy

*Geon-Hui Lee†, Cheonhoo Jeon†, Jee Won Mok, Sangbaie Shin, Su-Kyoung Kim, Hye Hyeon Han, Seong-Jong Kim, Sang Hoon Hong, Hwanhee Kim, Choun-Ki Joo, Jae-Yoon Sim, and Sei Kwang Hahn****

*Geon-Hui Lee, Su-Kyoung Kim, Hye Hyeon Han, Seong-Jong Kim, Sang Hoon Hong, Sei Kwang Hahn****
Department of Materials Science and Engineering, Pohang University of Science and Technology (POSTECH), 77 Cheongam-ro, Nam-gu, Pohang, Gyeongbuk 37673, Korea.
E-mail: skhanb@postech.ac.kr.

Cheonhoo Jeon, Jae-Yoon Sim
Department of Electrical Engineering, Pohang University of Science and Technology (POSTECH), 77 Cheongam-ro, Nam-gu, Pohang, Gyeongbuk 37673, Korea.

Jee Won Mok, Choun-Ki Joo
Department of Ophthalmology and Visual Science, Seoul St. Mary's Hospital, College of Medicine, The Catholic University of Korea, 505, Banpo-dong, Seocho-gu, Seoul 06591, Korea.

Sangbaie Shin, Hwanhee Kim
PHI BIOMED Co., 168, Yeoksam-ro, Gangnam-gu, Seoul 06248, Korea.

Supporting Display Items

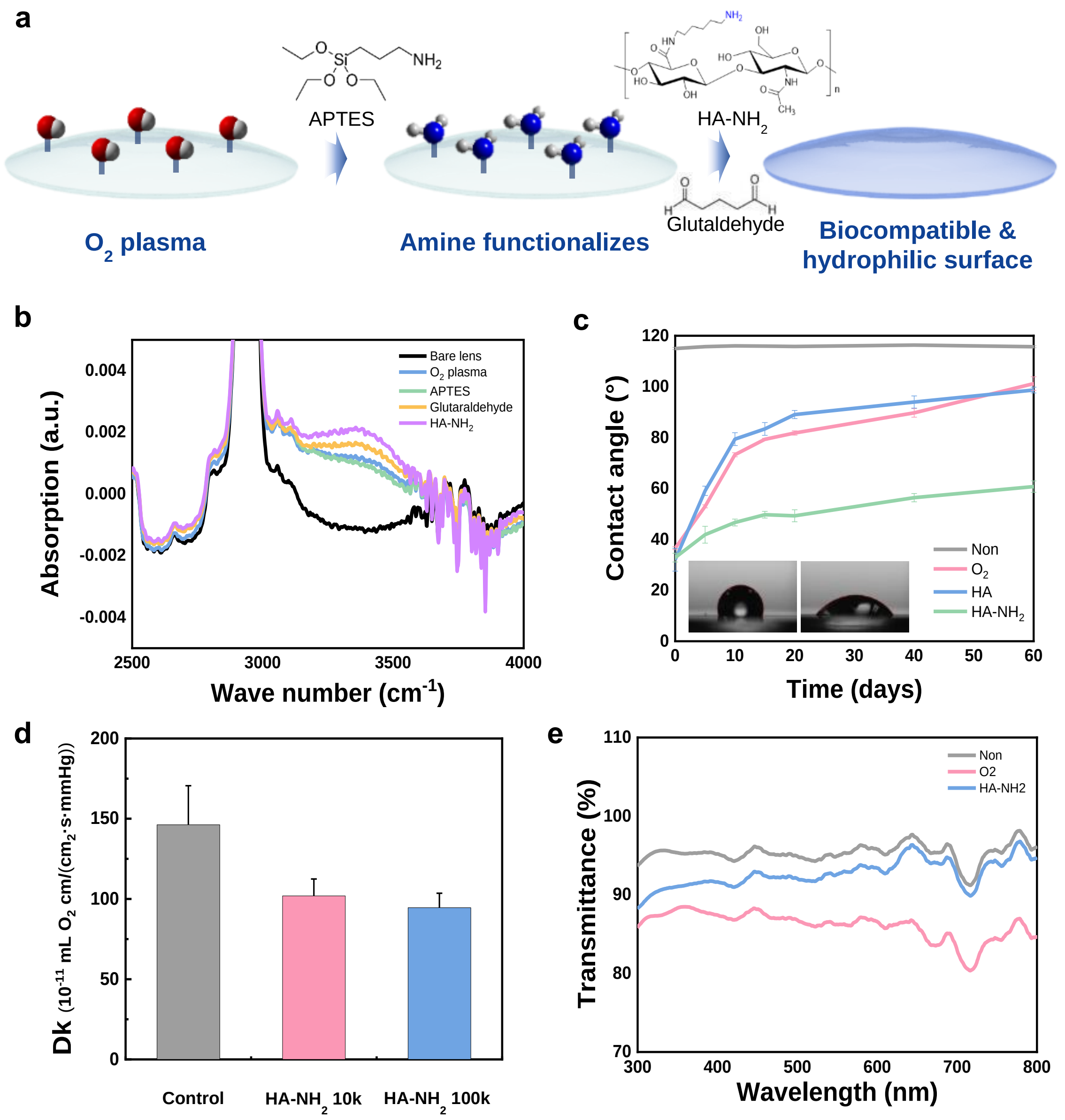

Figure S1 | Schematic illustration and characteristics of surface-modified contact lenses. **a,** Schematic illustration for the contact lens surface modification process. **b,** FT-IR analysis according to the contact lens surface treatment process. **c,** Contact angle change of contact lenses according to the surface treatment method. **d,** Oxygen permeability after surface treatment of contact lenses with HA-NH_2. **e,** Transmittance change with increasing wavelength according to the surface treatment method.

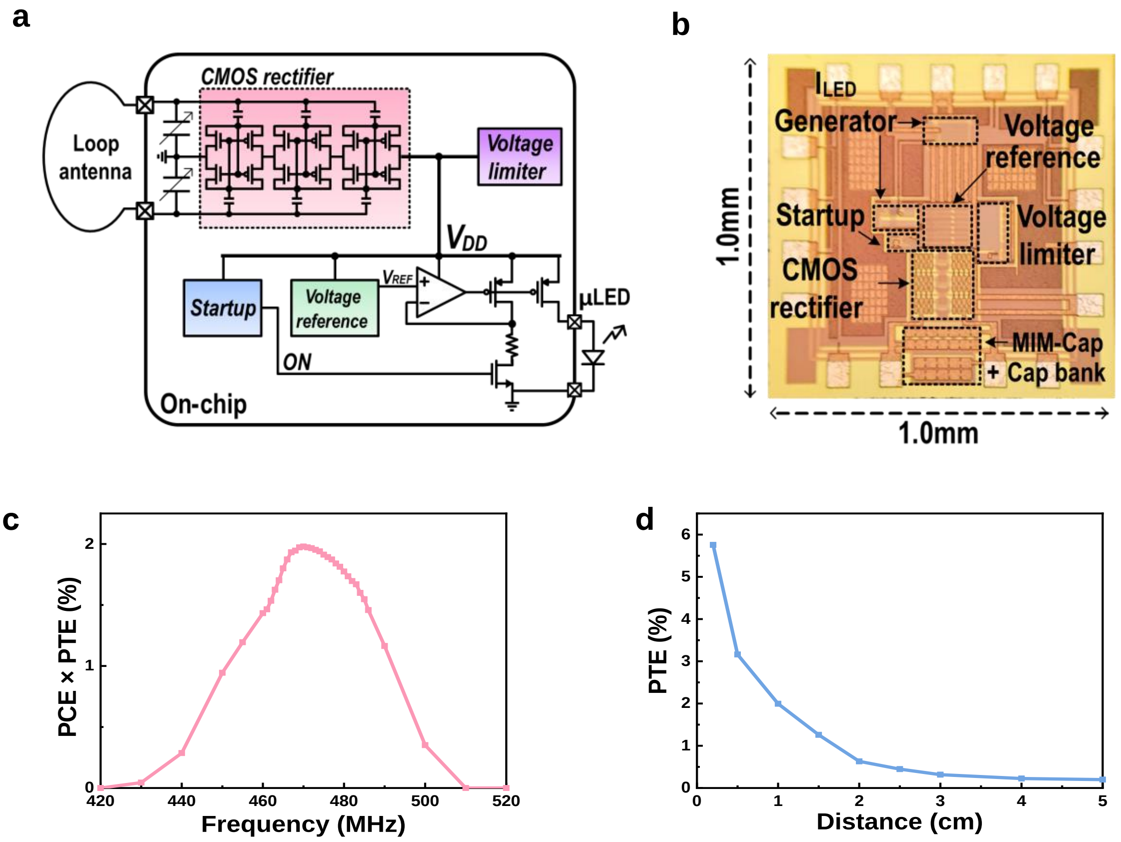

Figure S2 | Characteristics of wireless energy transfer. a, Schematic illustration of ASIC chip. **b**, OM image of ASIC chip. **c,** Power transfer efficiency (PTE) and power conversion efficiency (PCE) with increasing frequency change. **d,** PTE with increasing distance.

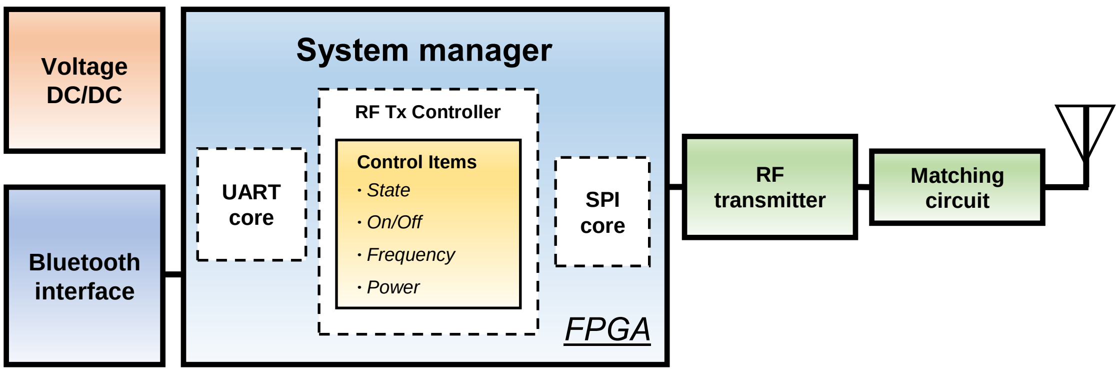

Figure S3 | Block diagram of pulse width modulation (PWM) circuit.

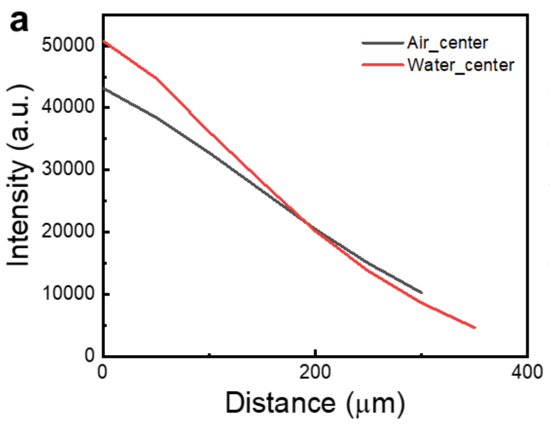

Figure S4 | The light intensity of far red/NIR micro-LED contact lens in water and in air by using an optical fiber. a, Center part of the contact lens. **b,** Edge part of the contact lens.

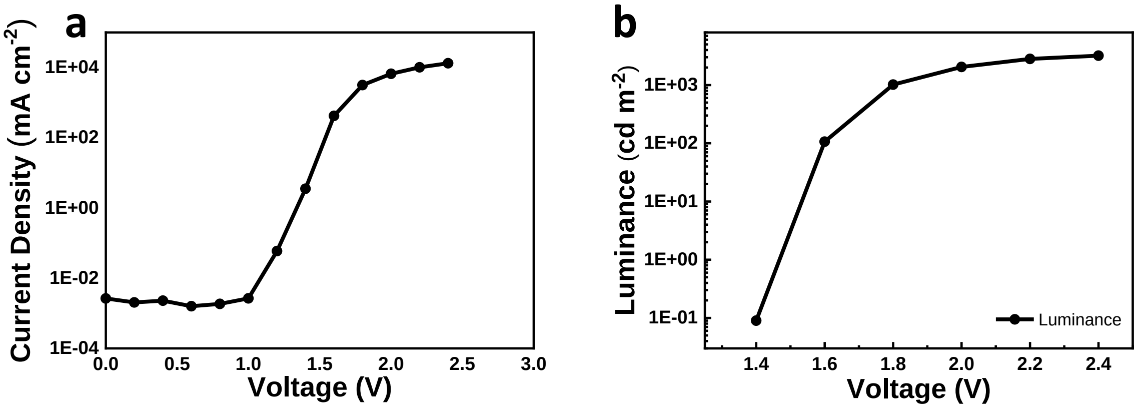

Figure S5 | Current density and luminance of far red/NIR micro-LED. a, Current density with increasing voltage applied to micro-LED. **b,** The luminance of micro-LED with increasing voltage.

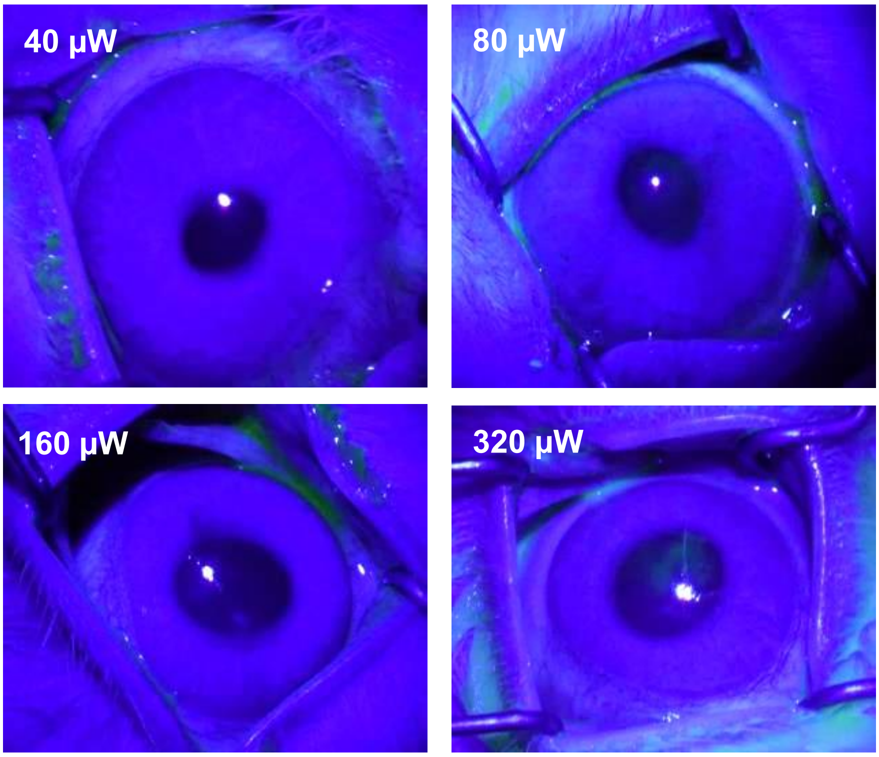

Figure S6 | Cornea safety shown by fluorescein staining.

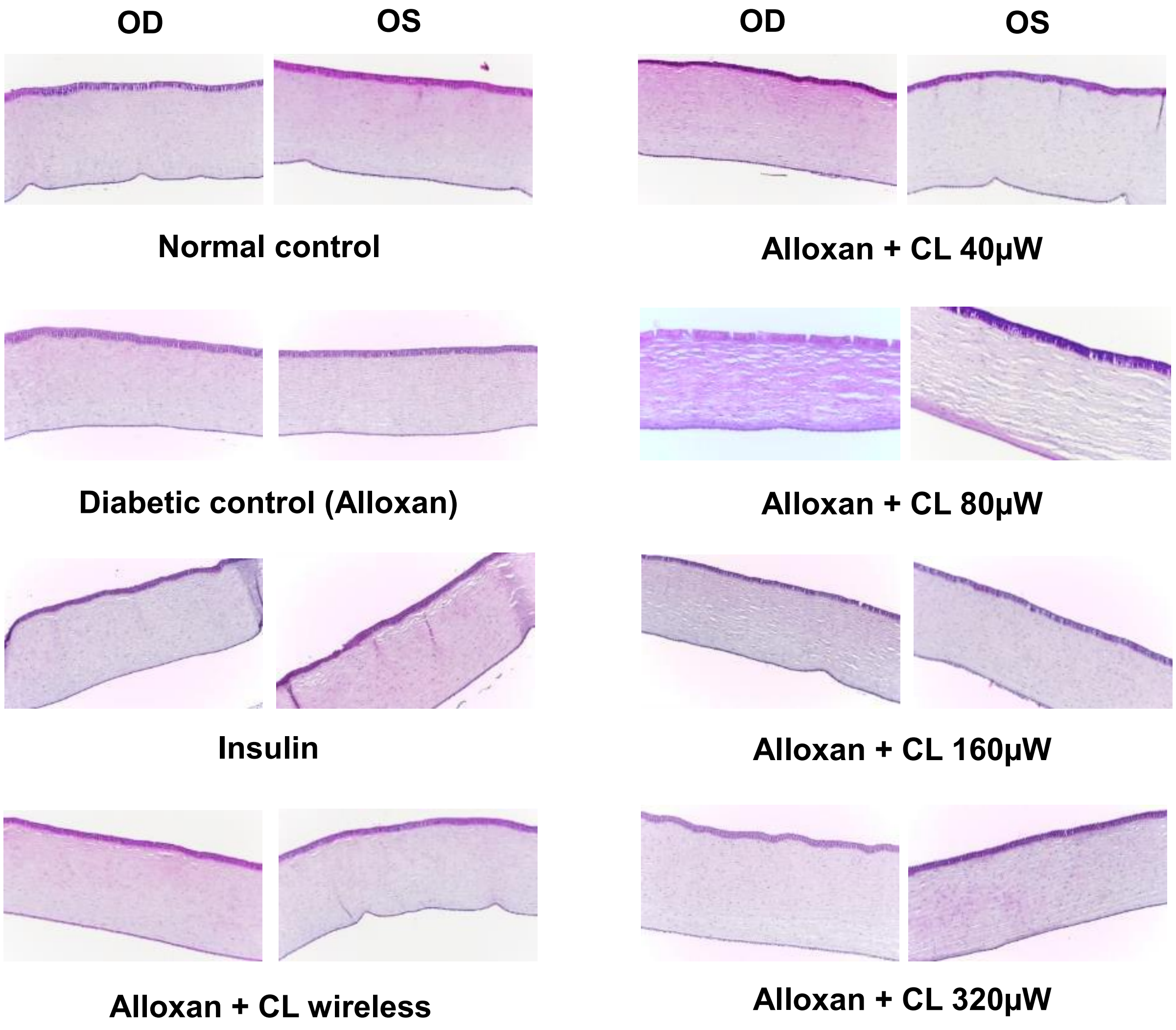

Figure S7 | Optical microscopic images for the assessment of corneal thickness.

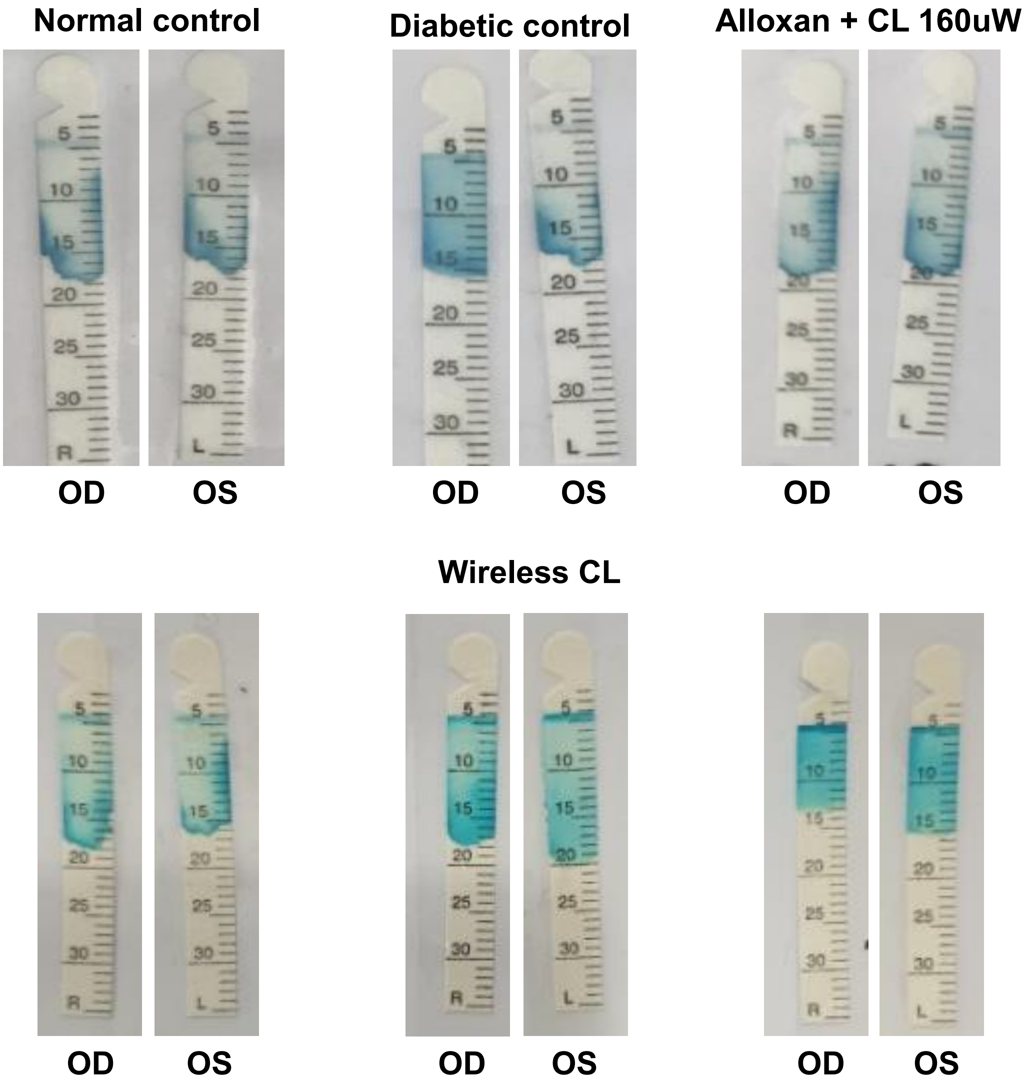

Figure S8 | Tear volume assessment by Schirmer's test.

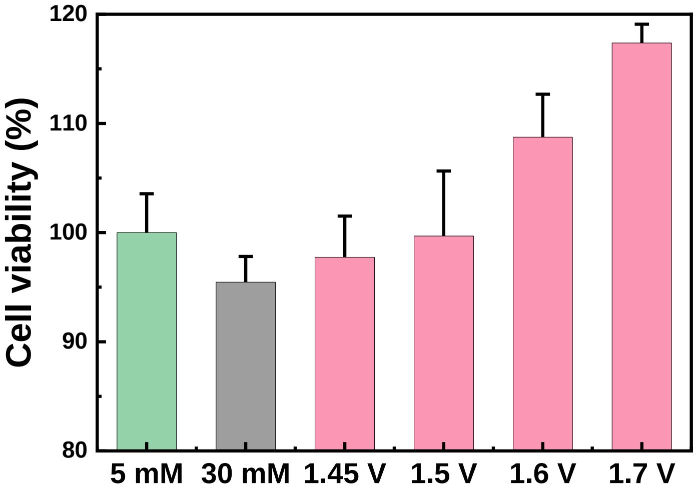

Figure S9 | Cell viability with increasing voltage for light (670 nm) intensity under the high glucose (30 mM) environment (n = 5).

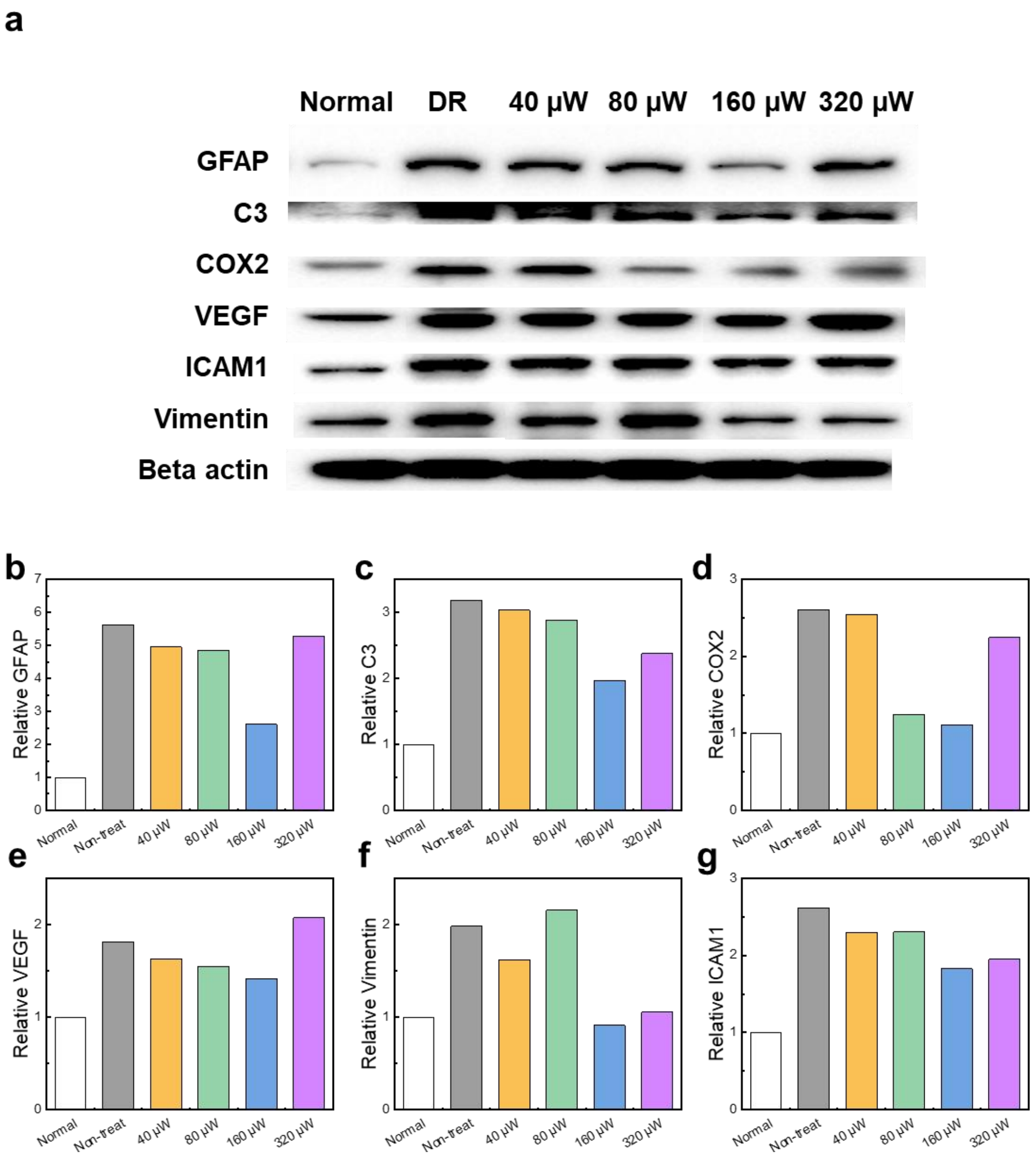

Figure S10 | a, Western blot image and analysis for the expression of **b,** GFAP, **c,** C3, **d,** COX2, **e,** VEGF, **f,** vimentin, **g,** ICAM-1 and β-actin as a control in the retina after treatment with wired LED contact lenses for 8 weeks.

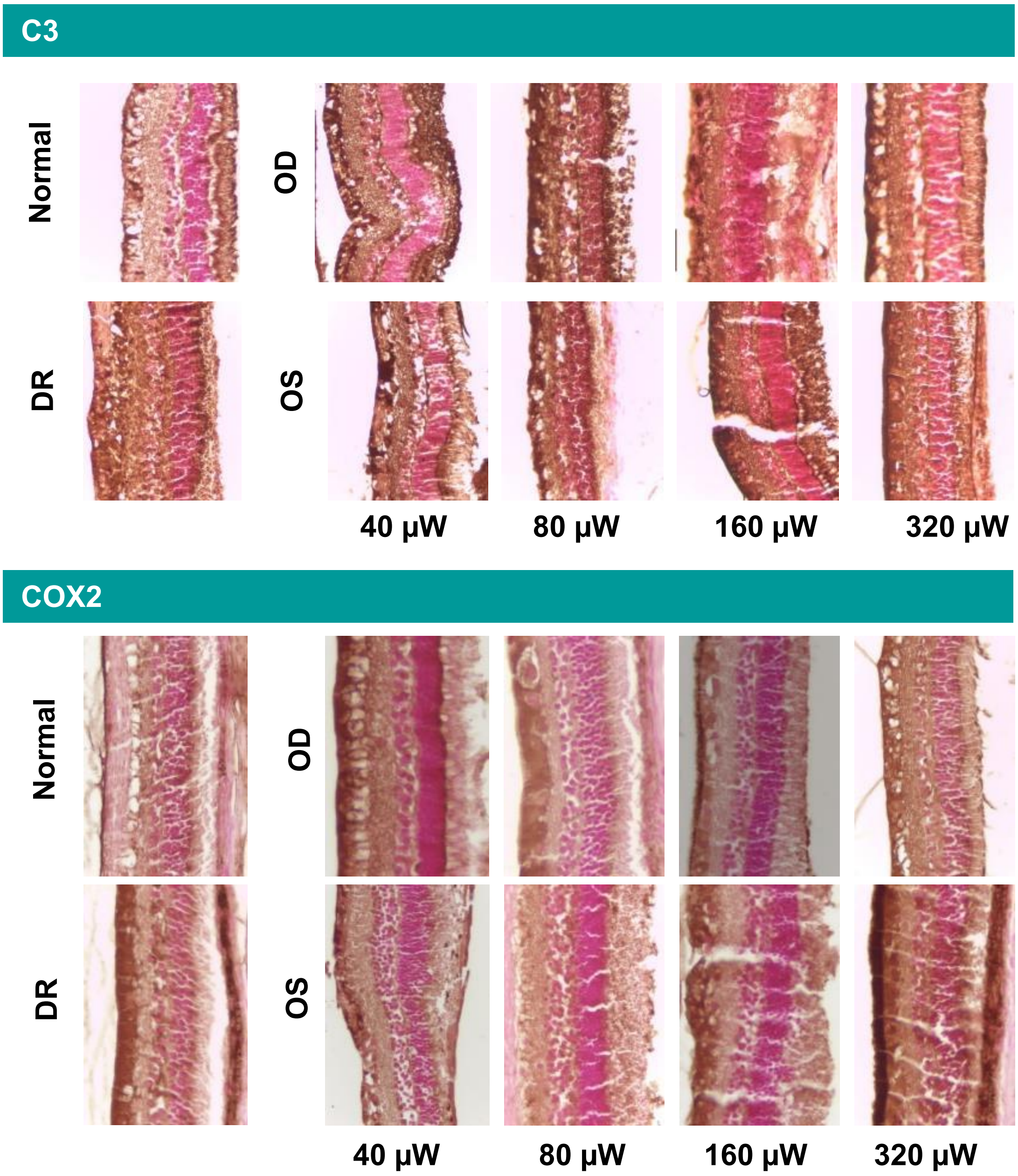

Figure S11 | Immunohistochemical images for C3 and COX2 according to the light intensity (40, 80, 160, and 320 μW).

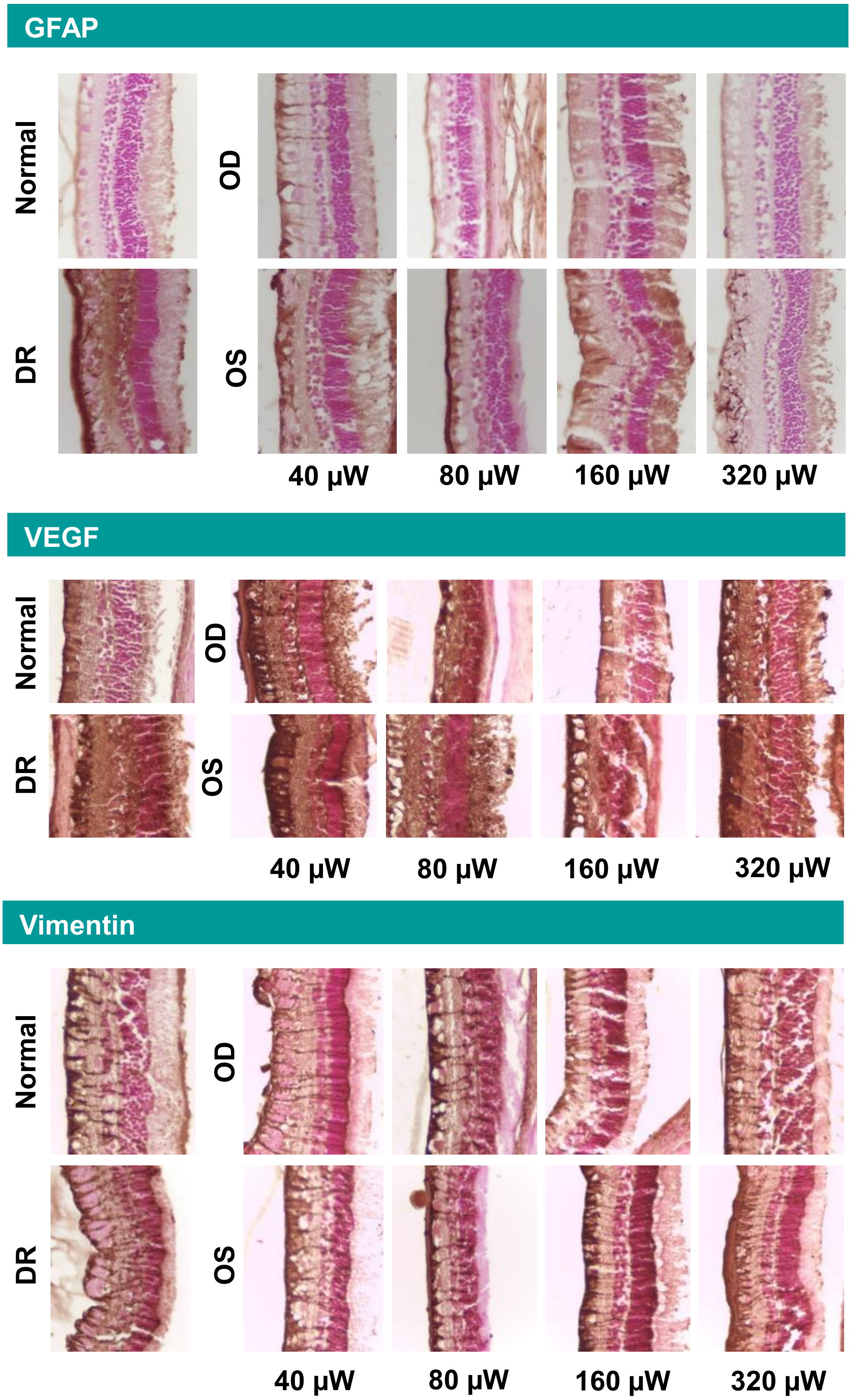

Figure S12 | Immunohistochemical images for GFAP, VEGF, and vimentin according to the light intensity (40, 80, 160, and 320 μW).

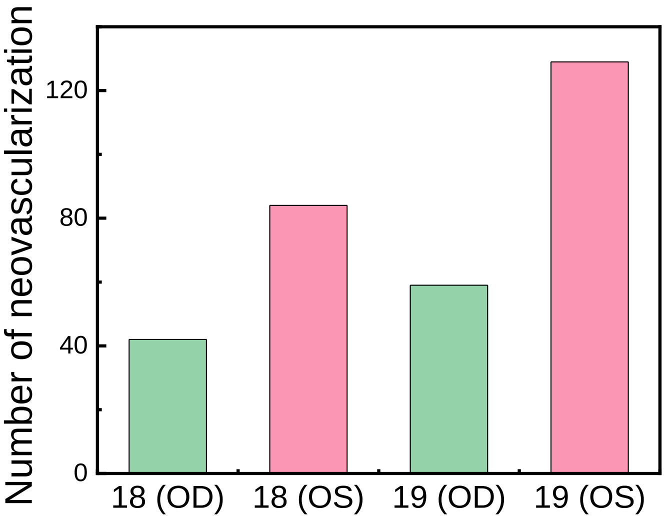

Figure S13 | The number of neovascularization and hemorrhage site.

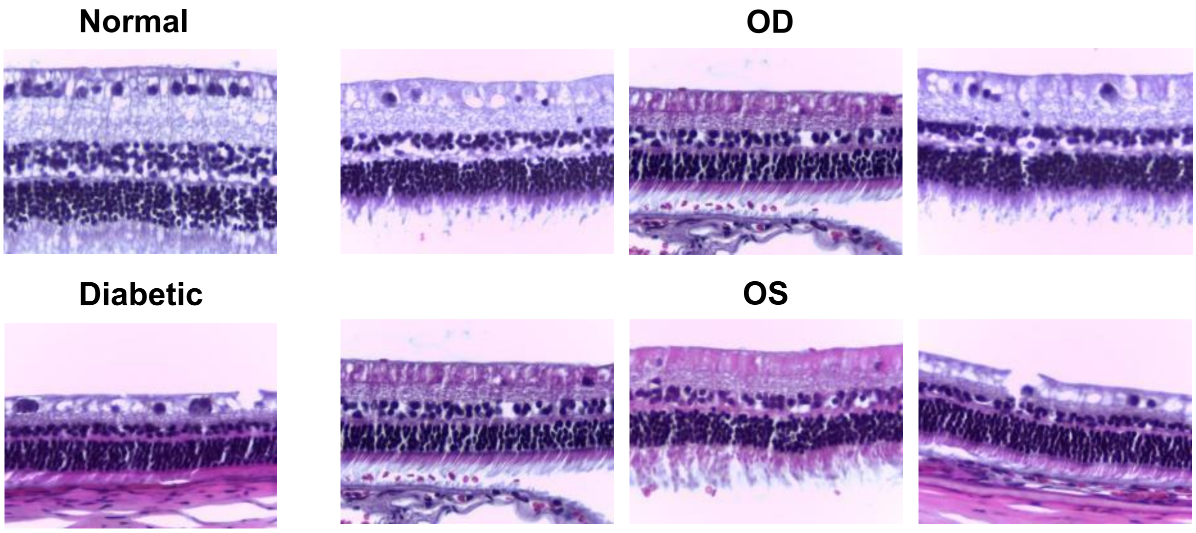

Figure S14 | The retina thickness of diabetic rabbits (OD) with and (OS) without wireless LED contact lens treatment (n = 3).

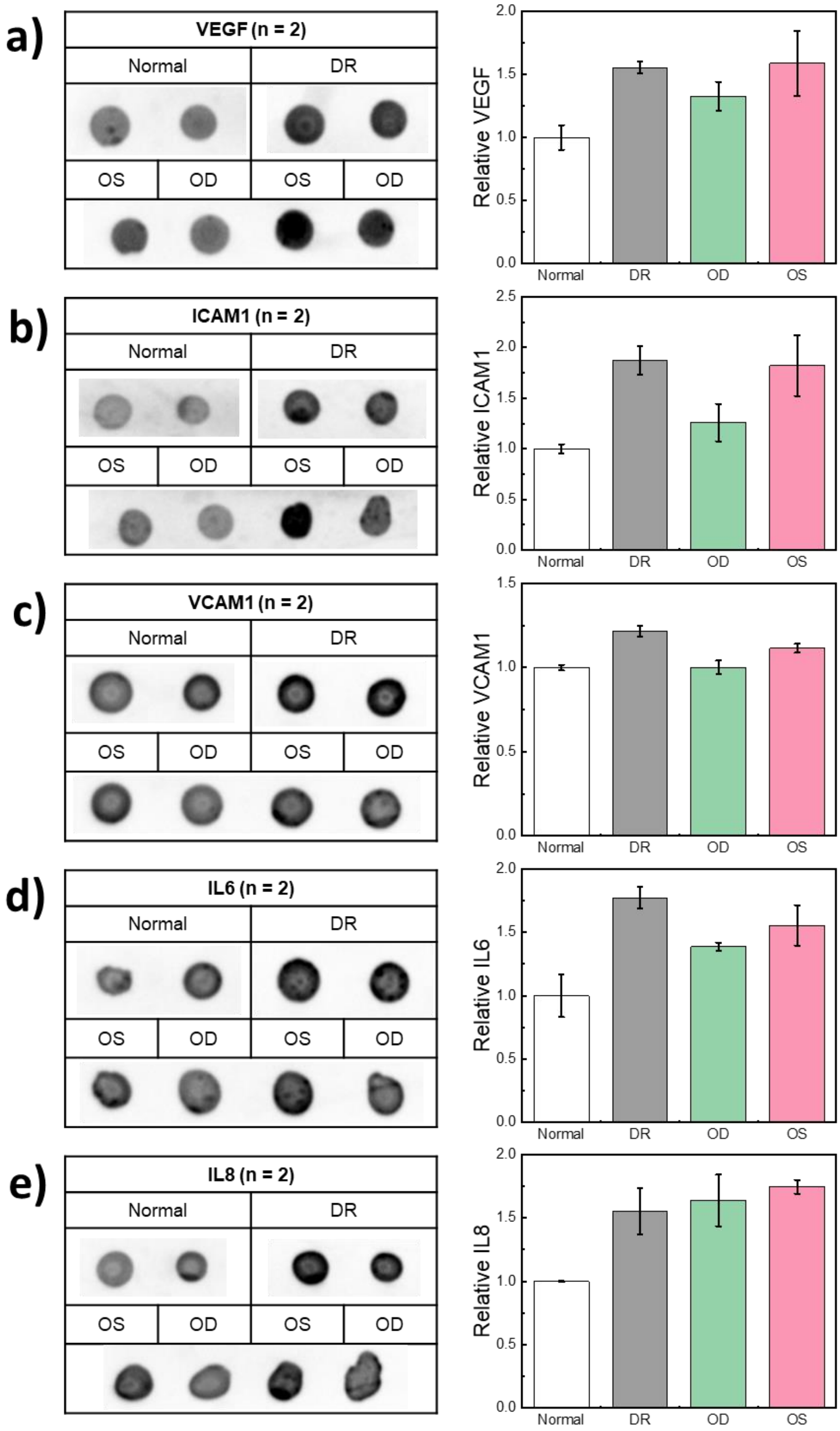

Figure S15 | Dot blot analysis of a) VEGF, b) ICAM-1, c) VCAM-1, d) IL-6, and e) IL-8 in the vitreous for the normal, diabetic retinopathy (DR), and the treated groups of OS and OD with wireless LED contact lenses for 8 weeks (n = 2).

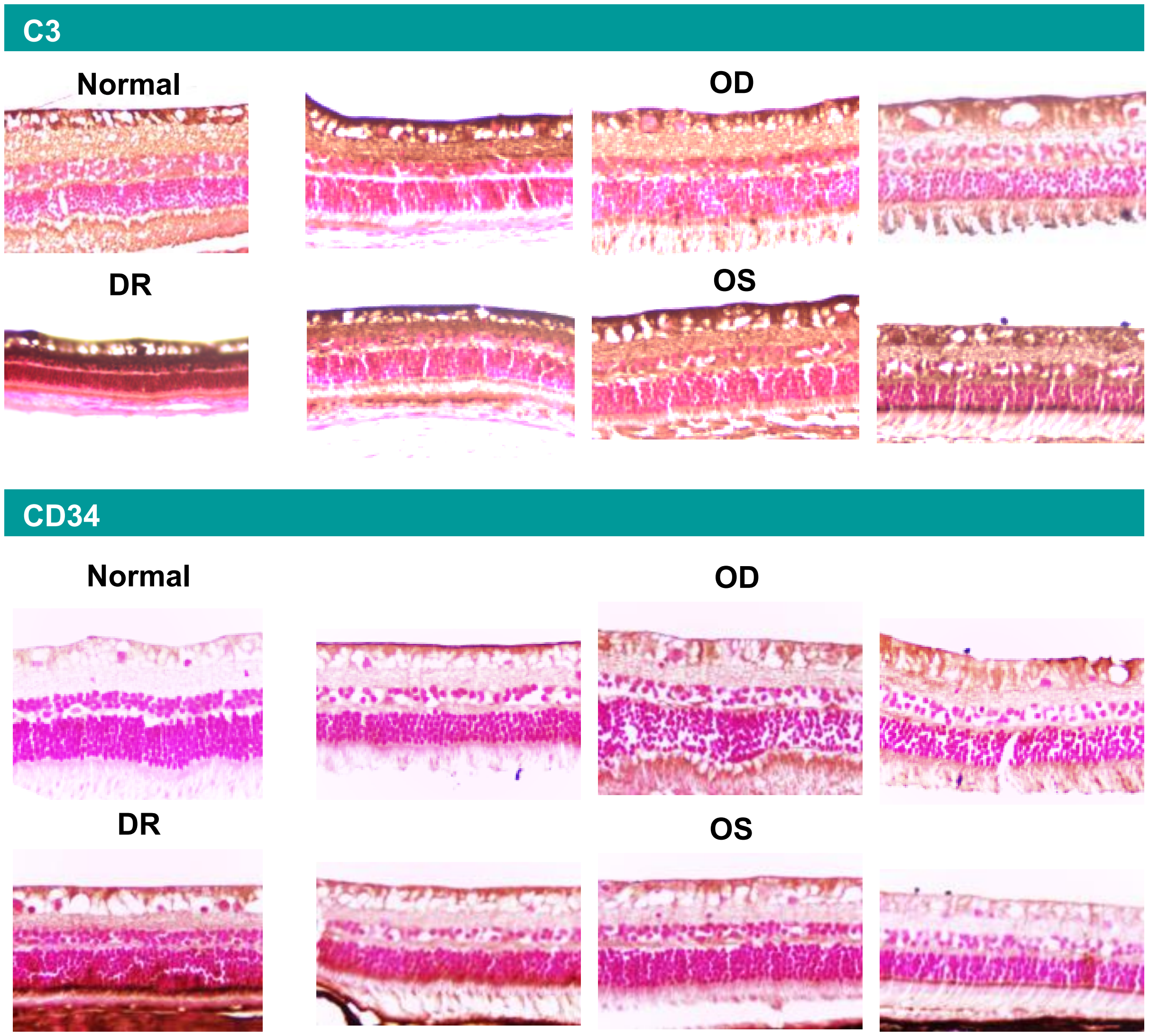

Figure S16 | Immunohistochemical images for C3 and CD34 after treatment of wireless LED contact lenses (light intensity: 120 μW).

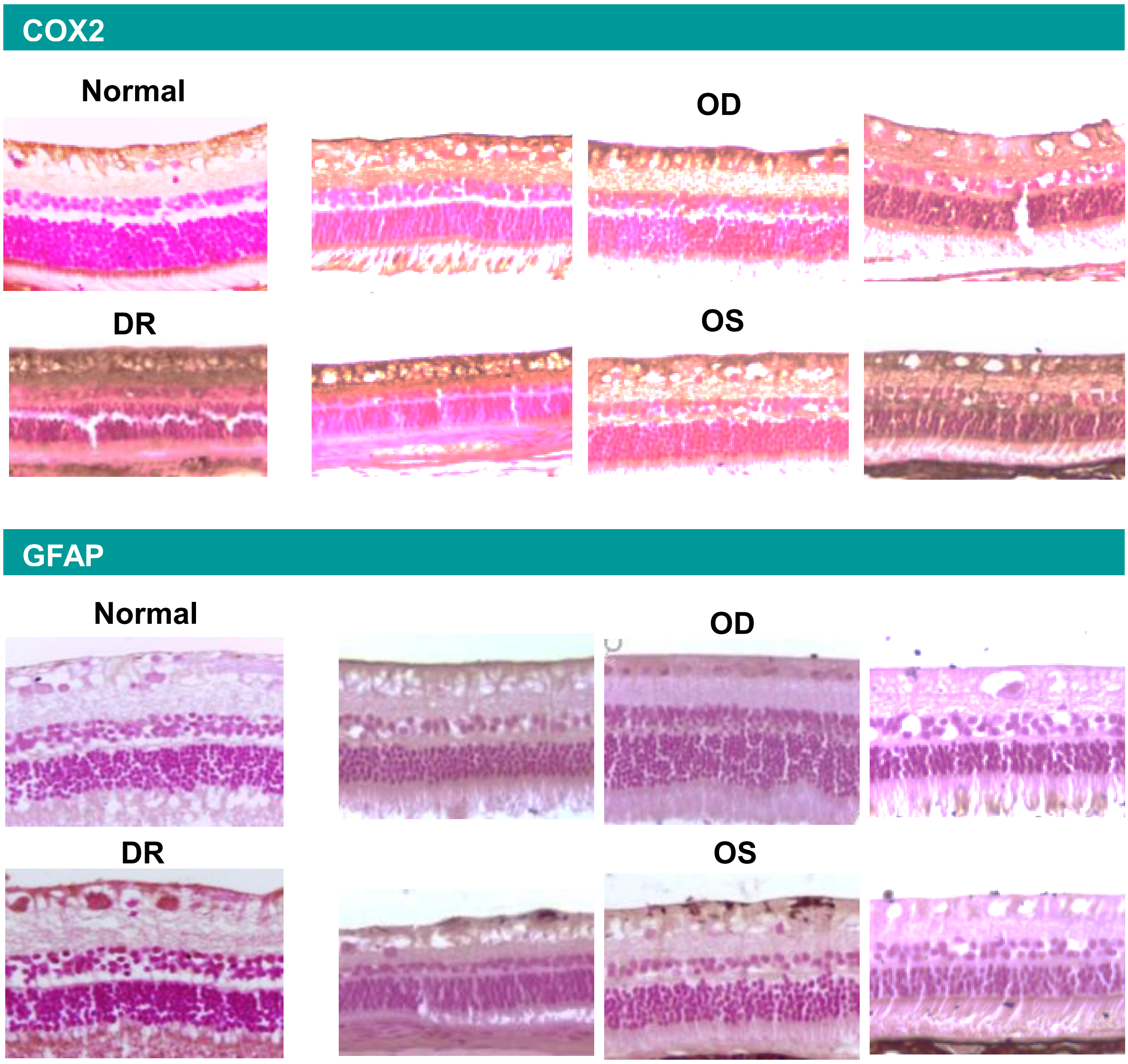

Figure S17 | Immunohistochemical images for COX2, GFAP and ICAM after treatment of wireless LED contact lenses (light intensity: 120 μW).

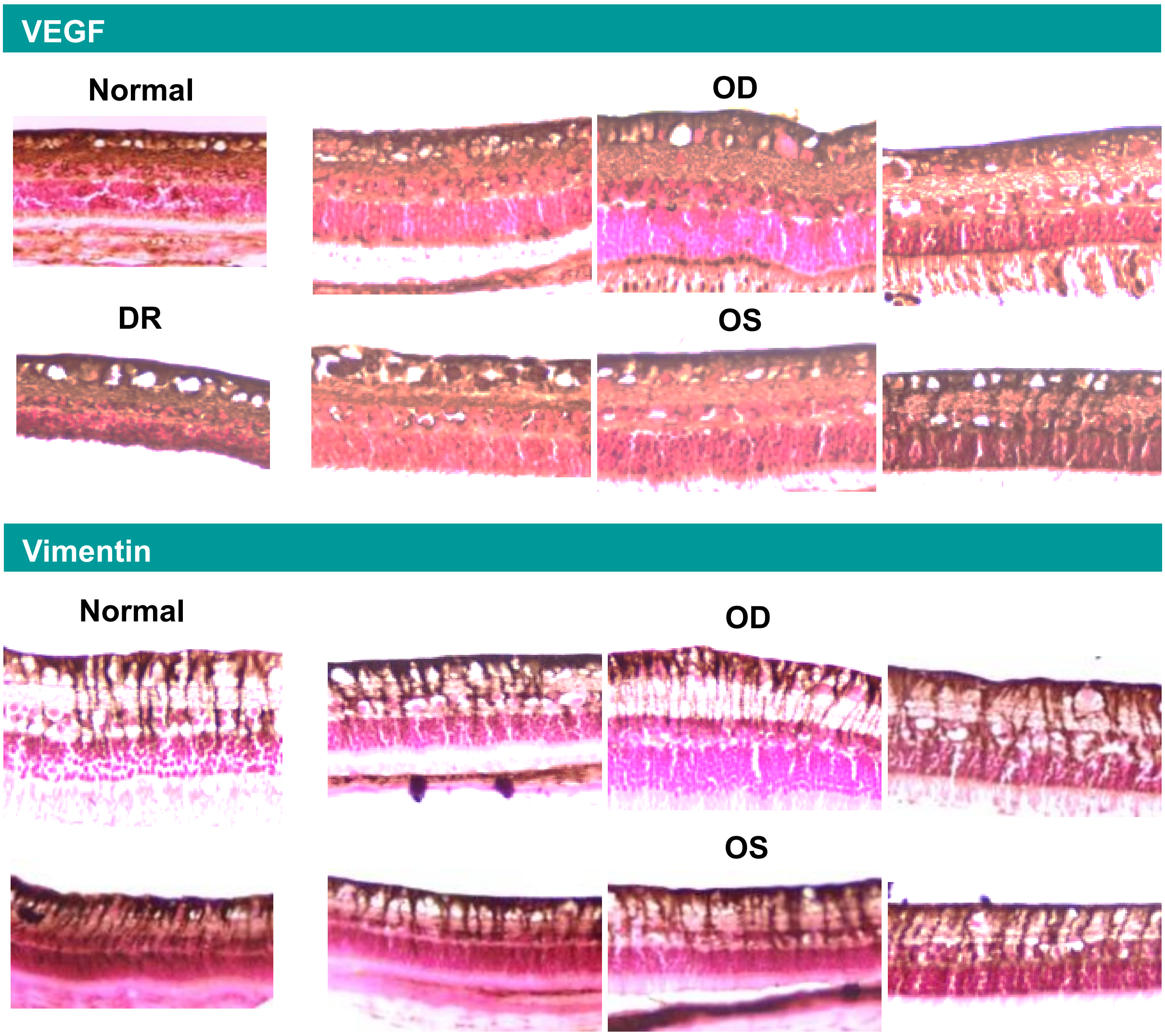

Figure S18 | Immunohistochemical images for VEGF and vimentin after treatment of wireless LED contact lenses (light intensity: 120 μW).

Table S1 | The purpose, pros and cons of surgery, laser treatment, and LED contact lens.

	Purpose	Pros	Cons	ref
Surgery	Treatment of secondary complications	• Clear vision recovery<br>• Neovascular growth factors reduction<br>• Surgically reattachment of detached retinas	• Severe complications by vitreous surgery in diabetic eyes<br>• Secondary complications treatment of a primarily microvascular disease<br>• Possibility of retinal ischemia and the increased neovascular stimulus	[1]
Laser treatment	Photocoagulation treatment of neovascular site	• Significant decrease of the proliferative retinopathy and macular edema	• Painful treatment<br>• Moderate visual loss by restricting the visual fields and nyctalopia<br>• Other side effects including glare, exudative retinal detachment, elevated intraocular pressure, and retinal fibrosis.	[2,3]
LED contact lens	Photobiomodulatory prevention of neovascularization	• Preventing the cause of diabetic retinopathy<br>• Patient compliance<br>• On-demand daily life application	• Glare while using LED contact lens<br>• Minimal effect on the severe diabetic retinopathy	This work

[1] H. Helbig, Surgery for diabetic retinopathy. *Ophthalmologica* **2007**, 221, 103-111.
[2] C. C. Bailey, J. M. Sparrow, R. H. B. Grey, H. Cheng, The national diabetic retinopathy laser treatment audit III. Clinical outcomes. *Eye* **1999**, 13, 151-159.
[3] G. E. Lang, Laser Treatment of Diabetic Retinopathy. *Diabetic Retinopathy* **2007**, 39, 48–68.
